# Ketamine for mental health - A naturalistic inventory of prescribing practices, safety, and adverse effects

**DOI:** 10.1371/journal.pmen.0000215

**Published:** 2025-04-02

**Authors:** Maegan Stuart, Ian Stefanuk, Rebecca Comeau, Atul Khullar, Jennifer Swainson

**Affiliations:** 1 Northern Ontario School of Medicine, Sault Ste. Marie, Ontario, Canada; 2 Department of Psychiatry, University of Calgary, Calgary, Alberta, Canada; 3 Department of Psychiatry, University of Alberta, Edmonton, Alberta, Canada; West China Hospital of Sichuan University, CHINA

## Abstract

Ketamine is an NMDA receptor antagonist, long used for its anesthetic and pain reducing properties, but has more recently demonstrated efficacy for treating depression. Evidence is also emerging for other psychiatric indications including posttraumatic stress disorder, anxiety disorders, obsessive compulsive disorder, and to support psychotherapy. In this context, there is greater demand for its use in various forms including intravenous, intramuscular, subcutaneous, intranasal, oral, and sublingual administration. While intravenous administration typically requires administration and monitoring in a healthcare setting, the safety precautions for other forms of ketamine are unclear. Limited data in this regard may elicit controversy among prescribers, who wish to improve patient access, and among regulatory bodies, who may impose limitations based on IV ketamine data. This project aims to bridge the gap between current literature and the clinical experience and opinions of ketamine prescribers. This information was obtained in the form of a survey and answered: “How do prescribers of ketamine, with emphasis on psychiatric care providers, find its safety profile and in what ways have they prescribed ketamine?” Information was gathered on prescriber profiles, indications for ketamine use, doses and routes of ketamine prescribed, side effects and adverse events, and prescribers’ opinions regarding monitoring requirements. Results were obtained from 45 providers with more than 1000 patient encounters over two countries. Non-IV forms of ketamine were commonly used, and the survey reported a favorable safety profile for ketamine. Non-IV Ketamine was reported to be safely utilized in a clinic setting with minimal monitoring and safety equipment, or at times, for home use. While survey results carry limitations, it appears that safety is dependent on dose and route, and strict universal regulations for all formulations may unnecessarily limit patient access.

## Introduction

Ketamine is an NMDA receptor antagonist most widely used for anesthesia and treating pain [1], and is also gaining popularity and evidence for use in psychiatry. Intravenous (IV) ketamine at subanesthetic doses has now been recognized internationally as an effective treatment for treatment-resistant depression (TRD) [2], which is classified as depression that has failed to respond to two or more antidepressant trials. Intranasal (IN) esketamine, an enantiomer of ketamine, has been approved in a number of countries, including Canada and the United States for TRD. Though depression is the indication with the largest body of evidence, emerging uses of ketamine in psychiatry include treatment of posttraumatic stress disorder (PTSD) [3], anxiety, obsessive compulsive disorder, substance use disorders, bipolar affective disorder, and as an augment to psychotherapy [4].

 Ketamine-assisted psychotherapy (KAP) involves giving ketamine before, during, or after psychotherapy and is thought to enhance the therapeutic process. In low doses, ketamine has been shown to enhance neural plasticity [5], and may enhance suggestibility, both of which may make patients more receptive to insights and emotional processing [6]. Additionally, the dissociative state that can be induced by ketamine enables some patients to more easily access and confront traumatic experiences, potentially allowing more therapeutic interventions [7]. In the unique learning conditions of psychedelic-assisted psychotherapy, corrective information can be implemented into relaxed belief systems, and may lead to long-term symptom reduction [8]. Increasingly, ketamine-assisted psychotherapy is being explored as a novel approach to treat conditions such as treatment-resistant depression, posttraumatic stress disorder, anxiety disorders, and others.

While the efficacy of IV ketamine and IN esketamine pharmacotherapy is well documented, both have barriers to use, including lack of access, cost, and monitoring requirements to administer in a healthcare setting. While less data is available regarding dosing, safety, and efficacy of ketamine given by other routes of administration, the recently published Canadian Network for Mood and Anxiety Treatments (CANMAT) Guideline for depression designated “non-IV” ketamine as a potential 3rd line adjunctive treatment for depression. However, due to heterogeneous reporting in the literature, there were no comments or recommendations regarding safe or effective dose ranges of these forms [9].

A meta-analysis of RCTs [10] reported highly varied doses (0.1-1.0 mg/kg) in IV ketamine used for depression, though doses below 0.5 mg/kg have not been deemed effective [11]. Other parenteral routes of administration are lacking such data and no meta-analyses are available. A small pilot study (n=15) found that that doses of ketamine administered by IV (n=4), intramuscular (IM) (n=5), and subcutaneous (SC) (n=6) were comparable, and efficacy was noted in the dose range of 0.1-0.5 mg/kg, with SC administration described as the most tolerable [12]. A retrospective cohort study of IM ketamine found that doses of 0.3-2.15 mg/kg with a median of 4 treatments improved both depression and anxiety by 38 and 50% respectively [13]. A study protocol looking at 0.75 mg/kg IM ketamine compared to escitalopram and aripiprazole has been published, but the study itself has not been completed [14]. A pilot study (n=10) [15] demonstrated that SC ketamine at a dose of 0.25-0.75 mg/kg used for a maximum of 6 treatments twice per week was well tolerated and effective in relieving symptoms of treatment resistant depression. Similarly, a pilot study (n=14) of SC ketamine in geriatric patients found that half of patients remitted, and 5/7 of these were at doses lower than 0.5 mg/kg. Despite efficacy data from these pilot and retrospective studies, optimal dosing for SC and IM ketamine remains unclear. Based on the more robust RCT data from IV ketamine, it could be expected that doses required would be similar or higher than for IV, as both SC and IM have slightly lower bioavailability than IV.

As for non-parenteral forms of ketamine, meta-analysis of IN ketamine [10] has identified 3 RCTs of IN esketamine and only one of IN ketamine. Both were deemed effective and doses used ranged from 28-84 mg for esketamine and 50 mg for the IN ketamine trial [16]. Protocols also varied with the usage of intranasal esketamine with doses from 28-84 mg and frequency from once per week to twice per week [17]. Despite the official approval of IN esketamine, use of IN ketamine itself remains unclear and perhaps variable among patients and devices. While only one RCT exists, interestingly, a pilot RCT of 10 patients was aborted early due to lack of tolerability at a 100 mg dose [18]. Contrasting this, retrospective chart reviews have noted efficacy and tolerability at doses 100-150 mg once or twice per week [19,20].

A more recent meta-analysis looking specifically at oral (PO) and sublingual (SL) ketamine administration included 22 studies, of which only 4 were randomized controlled trials [21]. The RCTs used regimens of 1 mg/kg by PO three times weekly for 3 weeks [22,23], 50 mg PO daily [24], and 150 mg PO daily [25]. While PO ketamine appeared safe and effective for depression, the non-RCT studies varied significantly in both dose range (0.5-1.25 mg/kg and 50-150 mg) and frequency of administration (daily to monthly) [10,21], making it difficult to determine optimal use of this formulation when treating depression. An open label trial not included in the above meta-analysis reported that efficacy of SL ketamine started at a dose of 300 mg and increased as tolerated to 450 mg as part of a telehealth KAP program. These patients used their ketamine at home, and no significant adverse events were noted in the large sample of over 1200 patients [26]. A retrospective study [27] used doses of sublingual ketamine from 0.5-1 mg/kg every 7-14 days.

Despite early promising results for efficacy, overall evidence to guide optimal dosing and frequency of non-IV forms of ketamine is significantly lacking, and there is a disconnect between what is currently available in the literature and the first hand anecdotal accounts of providers of ketamine. Similarly, there exists little data in the literature regarding safety of these formulations, and therefore unclear monitoring requirements to ensure safe administration. Because regulatory bodies in Canada and the United States have required both IN esketamine and IV ketamine to be delivered under observation in a health care setting [11,28], the default is to require the same monitoring for other formulations. In Canada, physician regulatory bodies in each province have set out to develop regulations for ketamine use. Some of these initial versions at the time this study was conceptualized were considered unnecessarily stringent by prescribers experienced with ketamine, yet there was little data to provide to support clinical experience in advocating for less stringent regulations.

As such, this project was conceptualized out of an online ketamine Google group that had formed with ketamine prescribers across Canada to advocate for appropriate regulations that did not unnecessarily restrict patient access to non-IV formulations. The aim of this project was to bridge the gap between available literature and anecdotal clinical experience, to better understand safety precautions needed for non-IV forms of ketamine.

With this information, regulatory bodies will have more data on safety profile and clinicians will have a greater understanding of dosing options for each formulation.

## Methods

Study participants were initially recruited via convenience sampling through a closed Canadian/American ketamine-focused professional interest group hosted on the Google Groups platform with a total of 250 group members. Subsequent participant recruitment was via snowball sampling as participants were encouraged to share the study survey with other academic or community colleagues prescribing ketamine. To improve reach, the survey was sent out on two separate occasions. Individual responses were reviewed and duplicate responses excluded.

Inclusion criteria were consent to participate in the study, any lifetime history of prescribing ketamine for any mental health indication, and practice location within Canada and/or the United States (to maintain ethics standards given variability in ketamine prescription regulations across jurisdictions). Individuals who prescribed ketamine exclusively for non-psychiatric uses such as pain control/anesthesia were excluded, but those who reported ketamine prescription for both mental health and non-mental health indications were included.

An anonymous web-based survey, using Google Forms, was sent to eligible ketamine prescribers in both Canada and the United States from May 31, 2021 to July 31, 2021. This was a qualitative survey and gathered background data about the prescribers, formulations used, dose ranges, safety profile, adverse events, suggested safety measures, and side effects of ketamine.

Members of the ketamine-focused professional interest Google Group were invited to provide feedback on the scope, relevance, and phrasing of a draft survey. Survey questions were modified and finalized using group-directed feedback prior to distribution. A copy of the survey can be viewed in S1 Appendix. This study received REB approval through the Laurentian University ethics board.

## Results

### Respondent characteristics

A total of 45 respondents completed the survey, including 16 respondents from Canada and 29 respondents from the United States. Of note, 38 respondents used ketamine in the context of psychotherapy. Prescribers noted a variable number of years in clinical practice with the largest category of ketamine prescribers having over 30 years in clinical practice. Table 1 reviews these demographics.

**Table 1:** Characteristics of ketamine prescribers.

Specialty	Number (% of respondents)
Psychiatry	24 (54.5%)
Family Medicine	9 (20.5%)
Emergency Medicine	5 (11.4%)
Anesthesiology	3 (6.8%)
Other	3 (6.8%)
**Years in practice**	**Number (% of respondents)**
0–5	3 (6.8%)
5–10	5 (11.4%)
10–15	8 (18.2%)
15–20	7 (15.9%)
20–30	5 (11.4%)
30+	16 (36.4%)
**Number of ketamine patients treated**	**Number (% of respondents)**
1–5	2 (4.44%)
5–10	4 (8.88%)
10–20	6 (13.33%)
20–30	8 (17.77%)
40–60	6 (13.33%)
60–80	4 (8.88%)
80–100	3 (6.66%)
>100	10 (22.22%)
>1000	2 (4.44%)
**Type of ketamine treatment**	**Number (% of respondents)**
Ketamine-assisted Psychotherapy	38 (84.44%)
Ketamine prescribed as pharmacotherapy	7 (15.55%)

### Duration of ketamine prescribing

**Fig 1:**
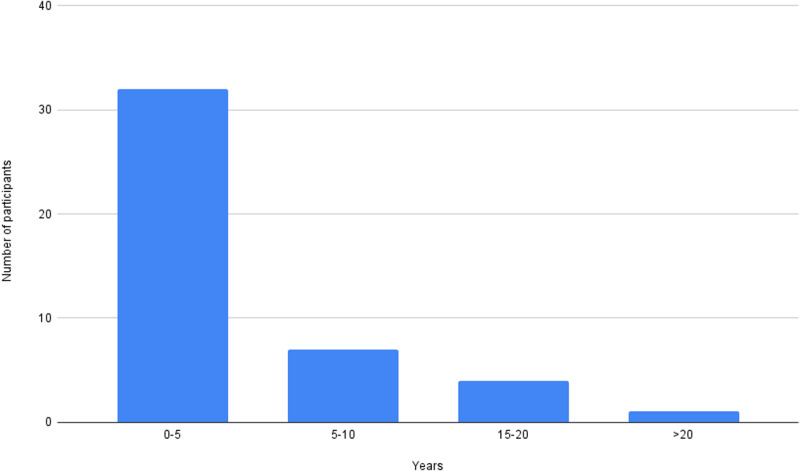
Duration of ketamine prescribing (in years).

The length of time that the providers have been using ketamine with their patients was quite variable, from 6 months to 32 years (Fig 1). The mean was 5.2 years, with the median experience being 2.5 years (Fig 1).

### Ketamine use characteristics

#### Indications for use.

A total of twenty three different indications for prescribing ketamine were reported. These included both psychiatric and non-psychiatric conditions, which may reflect the diverse specialties of the respondents. Of the 45 respondents, all used for psychiatric purposes, which were primarily depression (45) and PTSD (37). Less common psychiatric indications included suicidality (2), substance use disorders (2), OCD (6), end of life anxiety/palliative care (2), anxiety (6), dissociative identity disorder (DID) (1), autism (1), eating disorders (1), insomnia (1), and bipolar affective disorder (1).

A total of 20 respondents also used ketamine for non-psychiatric purposes including anesthesia (9), pain control (19), pruritus control (1), and complex regional pain syndrome (CRPS) (1).

### Administration route and dosing

**Fig 2:**
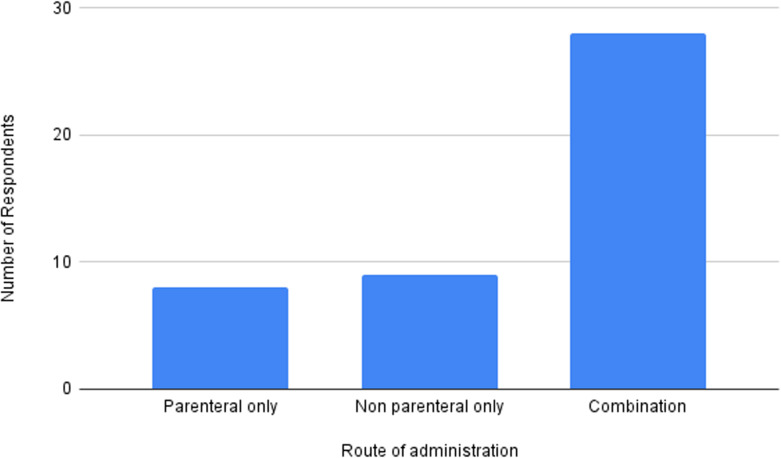
Distribution of parenteral and non-parenteral ketamine prescribed.

**Fig 3:**
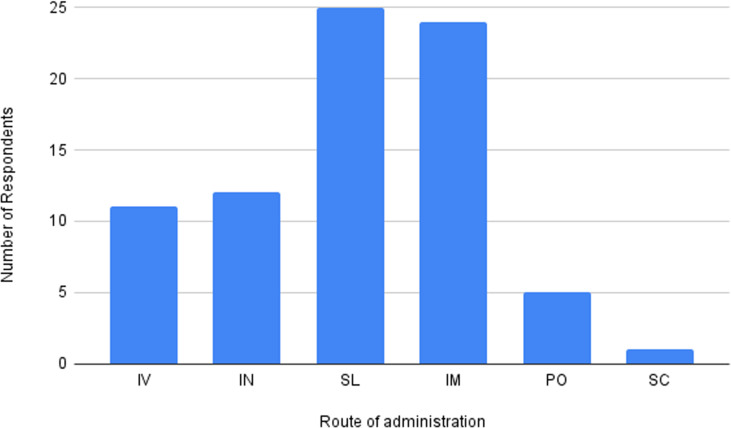
Distributions of all forms of ketamine utilized by survey respondents.

Of the 45 respondents, 28 providers used combinations of parenteral and non-parenteral routes, whereas 9 used non-parenteral only and 8 used parenteral only (Fig 2). IV ketamine was used by 16 respondents, but the most common parenteral route was IM (28) and one respondent reported SC use (Fig 3). Among the non-parenteral routes, SL administration was most popular (33), followed by IN (14), and PO (8).

#### Typical doses by route.

Some respondents provided the typical dose using a mg/kg factor, and others provided a range of absolute doses used (Table 2). Some respondents provided several different dosing ranges depending on indication for use and on patient characteristics, such as age. While most respondents did not include what dose they used for each indication specifically, doses that were clearly indicated for non-mental health purposes were removed. Table 2 summarizes prescribing practices including route and dose ranges, and Box 1 provides comments on dosing schedules provided by several respondents.

**Table 2:** Patterns of prescribing across ketamine formulations.

Dosing	IV	IM	SC	IN	SL	PO
Number of respondents using this formulation	16	27	1	14	32	8
Respondents favoring this route	9	22	0	1	10	1
Range of Patients treated	6-3000+	2-160	No data	4-100+	2-300	16-100
Mean (SD) patients treated	352 (880)	51 (54)	N/A	25 (30)	43 (63)	48 (48)
Mode patients treated	100	25	N/A	15	20	33
mg/kg range (number of respondents)	0.5-2.5 (13)	0.5-1.75 (12)	N/A	0.3-3 (3)	0.05-6.0 (7)	0.3-1.0 (1)
Absolute dose range(mg) (number of respondents)	50-600 (3)	20-600 (15)	30-45 (1)	30-250 (11)	50-800 (25)	50-800 (7)

**Box 1. Qualitative comments provided regarding dosing**

- "IM range 75-160 mg average 110"
- "IN used only to titrate the SL dose (10 mg/insufflation) 2-4 sniffs; SL range 50 - 300mg (average for relational experience 87,5 mg) (average for transformational experience 200 mg)"
- "Initial - start at 50-100mg SL and titration up as needed. Maintenance is what has been effective, and often less frequent"
- "Initial IM dose in office is usually between 0.5- 0.75 mg/kg. When response/sensitivity has been established, dosing is variable at 0.8-1.8 mg/kg."

#### Dose frequency.

**Fig 4:**
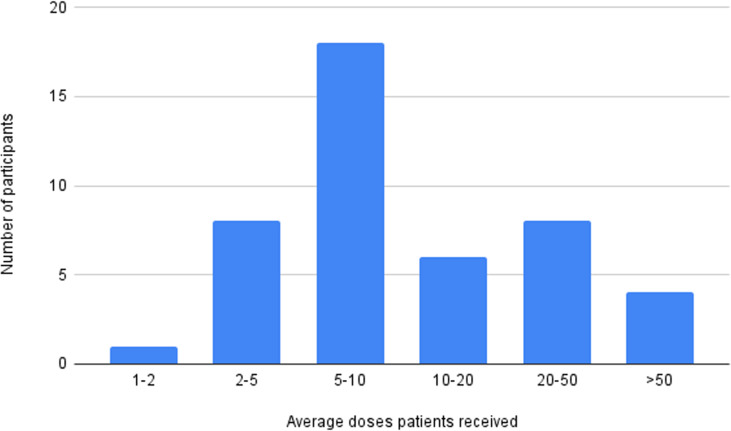
Average number of ketamine doses per patient.

All 45 respondents responded to the question regarding timing of the ketamine doses. 10 respondents answered with “weekly.” 5 respondents answered with “monthly.” There were 7 respondents for “twice weekly,” 4 responses for “variable,” 3 responses for “daily,” 2 responses for “single,” and 2 responses for “initial weekly, maintenance monthly.” Other respondents described treatment protocols with varying intervals. There were 10 responses that had similar treatment protocols - all began with twice weekly for 1-3 weeks followed by a maintenance phase which was variable from “weekly then monthly,” “every 2-3 weeks,” “every 3-4 months,” “every 1-6 weeks,” and “twice a year.” There were two additional responses, one for “3-4 times weekly” and “twice weekly to weekly.” On average, the majority of respondents stated that their patients receive on average between 5-10 doses. The next most common answers were between 2-5 doses and 20-50 doses (Fig 4).

### Location of prescribing

Ketamine was strictly prescribed in a hospital setting by only one respondent, who reported use of both SL and IV ketamine. All other 44 respondents used ketamine in an outpatient clinic setting. Over 80% of these respondents prescribed ketamine strictly in an outpatient clinic setting, while the remaining used a combination of hospital and clinic. Thirteen respondents commented that their patients self-administer the ketamine at home, and Box 2 provides narrative comments about home use, provided by 3 respondents. Table 3 reports distribution of prescribing locations by route of administration.

**Table 3:** Location of ketamine prescribing by route of administration.

Route of administration	Hospital only N (%)	Clinic only N (%)	Clinic and hospital N (%)
Sublingual (n=32)	1 (3.1%)	24 (75%)	7 (21.9%)
Intramuscular (n=28)	0	21 (77.8%)	6 (22.2%)
Intravenous(n=16)	1 (6.3%)	9 (56.3%)	6 (37.5%)
Intranasal (n=14)	0	10 (71.4%)	4 (28.6%)
Oral (n=8)	0	6 (75%)	2 (25%)
Subcutaneous (n=1)	0	1 (100%)	0

**Box 2. Qualitative comments provided regarding home ketamine use**

-“I train for at home use with SL doses from 150-300 mg. At home patients may use 50-450 mg SL, depending on prearranged individual protocols."
- “Am just starting to take home ketamine treatments so I don’t have a good feel for it yet. I am starting at much lower doses. In the 25 to 50 mg SL range"
- “Initial tx is generally always done in office while we dial in the dose and monitor how a patient responds. We normally start with a trial of 3 ketamine sessions, spaced differently depending on indication and need, before deciding on continuing the treatment plan. After initial sessions it could be possible for patient to begin at-home sessions if it is a burden to come into the office."
- “Initial = sl + IM. Maintenance is sl only, at home"

### Safety

#### Monitoring/interventions used.

The survey asked participants what type of interventions, monitoring, and safety measures they implemented pre and post treatment, and during treatment. Table 4 presents this data as reported by prescribers using parenteral, non-parenteral, and combination formulations of ketamine. No respondent reported a need to use these emergency safety measures.

**Table 4:** Ketamine monitoring and safety measures in place by prescriber type.

Safety measure/Intervention	non-parenteral prescriber only (n=9)	Parenteral prescriber only (n=8)	Combination prescriber (n=28)
Pre-Treatment Monitoring			
Cardiac History	9 (100%)	7 (87.5%)	28 (100%)
Seizure History	7 (77.8%)	5 (62.5%)	27 (96.4%)
Anesthetic History	3 (33.3%)	6 (75%)	21 (75%)
Basic Labwork	4 (44.4%)	0	8 (28.6%)
Blood Pressure	7 (77.8%)	7 (87.5%)	26 (92.9%)
Weight	4 (44.4%)	8 (100%)	21 (75%)
Other Vitals	5 (55.6%)	3 (37.5%)	13 (46.4%)
Allergies	9 (100%)	6 (75%)	24 (85.7%)
Psychiatric history	1 (11.1%)	2 (25%)	11 (39.3%)
Other history	0	1 (12.5%)	7 (25%)
ECG	1 (11.1%)	0	4 (14.3%)
Monitoring During Treatment			
BP	5 (55.6%)	7 (87.5%)	21 (75%)
HR	5 (55.6%)	6 (75%)	14 (50%)
RR	0	5 (62.5%)	15 (53.6%)
LOC	1 (11.1%)	2 (25%)	10 (35.7%)
Subjective Reports	7 (77.8%)	7 (87.5%)	16 (57.1%)
Emergency Measures in Place			
ACLS Trained staff	3 (33.3%)	7 (87.5%)	15 (53.6%)
AED	0	6 (75%)	13 (46.4%)
Oral airways	1 (11.1%)	3 (37.5%)	14 (50%)
Emergency Medications	3 (33.3%)	6 (75%)	20 (71.4%)
Staff present during administration			
MD	6 (66.7%)	7 (87.5%)	25 (89.3%)
Nurse	1 (11.1%)	7 (87.5%)	12 (42.9%)
Other	2 (22.2%)	2 (25%)	3 (10.7%)
Therapist	1 (11.1%)	1 (12.5%)	9 (32.1%)
NP	0	0	1 (3.6%)
None	2 (22.2%)	0	1 (3.6%)
Post-Treatment Monitoring			
None	1 (11.1%)	0	2 (7.1%)
15-30 minutes	0	2 (25%)	2 (7.1%)
30-60 minutes	2 (22.2%)	4 (50%)	10 (35.7%)
1-2 hours	1 (11.1%)	1 (12.5%)	9 (32.1%)
2 hours	1 (11.1%)	0	4 (14.3%)
As long as necessary	0	1 (12.5%)	1 (3.6%)
2-4 hours	3 (33.3%)	0	0

In follow up to asking respondents what safety measures were in place, an open ended question asked what specific measures they felt were actually necessary. Just over 25% (13) of respondents who used a variety of routes (4 IV, 3 IN, 7 SL, 6 IM, 2 PO) felt that no advanced safety measures were necessary. Overall, fewer prescribers who used only non-parenteral forms of ketamine reported that safety measures should be required, as compared to those who use parenteral and combined types of ketamine (Table 4). In particular, those who prescribed only non-parenteral ketamine did not indicate a need for a crash cart, oxygen, vital signs, presence of an AED, or ACLS training.

#### Adverse events associated with ketamine treatments.

Transient and mild adverse were common (Table 5), and occasionally were treated. Anti-nausea medications (ondansetron, metoclopramide, dimenhydrinate, or promethazine) were often used, sometimes prophylactically. Headaches usually resolved spontaneously but occasionally required over-the-counter (OTC) medications (ibuprofen or acetaminophen), and one patient required IV migraine medications before ketamine treatment. Hypertension was occasionally treated with antihypertensive medication. There were 4 reports of irritable bladder, but all noted the effect was also temporary, lasting hours to days following treatment.

**Table 5:** Side effects of ketamine (parenteral vs. non-parenteral).

Side effect	Non-parenteral only (n=9)	Parenteral only (n=8)	Combined (n=28)
Nausea/vomiting	3 (33.3%)	7 (87.5%)	18 (64.3%)
Headache	4 (44.4%)	4 (50%)	13 (46.4%)
Insomnia	1 (11.1%)	0	7 (25%)
Hypertension	0	3 (37.5%)	7 (25%)
Irritable bladder	2 (22.2%)	0	4 (14.3%)
Panic	1 (11.1%)	3 (37.5%)	10 (35.7%)
Dysrhythmias	0	2 (25%)	1 (3.6%)
Other	0	Dizziness, Nightmares, BV	SH, None, AB, Dissociation, A, PTR

* “Other” responses included n = 1 reports of: dizziness, nightmares, self-harm (SH), blurry vision (BV), aggressive behavior (AB), agitation (A), dissociation, prolonged trauma response (PTR).

The more potentially concerning side effects were not reported by respondents who used only non-parenteral forms of ketamine (Table 5). Among respondents who prescribed parenteral ketamine and the combination of parenteral and non parenteral, three reported transient dysrhythmias which resolved spontaneously. A fourth report indicated that a patient was discovered to have an pre-existing tachyarrhythmia and was treated with beta-blockers. Similarly, three episodes of behavioral adverse events were noted in non-mental health indications. One respondent who used SL or IM ketamine for anesthesia and pain control reported an episode of self harm which lasted 30 minutes and required restraints, but resolved spontaneously. Another respondent who used IM and SL ketamine for indications of anesthesia and/or pain control reported an adverse event of aggressive behavior, which lasted 25 minutes and required benzodiazepines with restraints. This prescriber reported using doses of 50-300mg, but it was unclear what dose and formulation was associated with this incident. Another prescriber who used IM (0.5-1.75 mg/kg), IN (30mg TID-QID), and SL (50-450 mg up to twice weekly) ketamine for pain indications noted one incident of 5 minutes of agitation, which resolved spontaneously. It was unclear which formulation or dose caused the incident. There was one response for prolonged trauma flashbacks, lasting 30 minutes and resolving with psychological support. There was one response each for dizziness (2 days) or nightmares (2 days). Side effects separated by mode of prescribing are listed below in Table 5.

With respect to long-term adverse effects, the majority of respondents (30, 68.88%) reported no long-term adverse effects. There were 10 respondents (22.22%) that noted tolerance to ketamine’s dissociative effects. Potential red flags for substance abuse were noted in 11 respondents and are noted in Table 6 below.

**Table 6:** Reported red flags for substance misuse/abuse.

Abuse related reports	Non-parenteral only (n=9)	Parenteral only (n=8)	Combined (n=28)
Cravings	0	1 (12.5%)	2 (7.1%)
Drug seeking behavior	1 (11.1%)	0	3 (10.7%)
Addiction	1 (11.1%)	2 (25%)	2 (7.1%)
Inappropriate Use of ketamine	0	0	2 (7.1%)

## Discussion

This project was undertaken with the aim to bridge the gap between the currently limited existing literature and anecdotal reports from providers regarding ketamine prescribing for mental health indications, particularly in regards to non-IV formulations, and further to non-parenteral formulations. Though IV ketamine has best evidence for mental health, primarily depression, access to IV ketamine treatment is limited in the public system and cost in a private setting is a barrier to many patients. The latest CANMAT depression guidelines [9] identify “non-IV” ketamine as potential 3rd line adjunct for patients with depression, but do not provide guidance for safe use due to limited evidence base. Advocates for ketamine accessibility have identified the potential for non-parenteral ketamine to be safely used by patients at home [29], but this concept is met with controversy. In this survey of ketamine prescribers, common practices are reported and, although they are not systematic reports of safety, can help inform clinical practice. While this study did report on some more traditional uses of ketamine such as anesthesia and pain control that would necessitate higher doses, respondents largely indicated that they used ketamine for mental health indications including depression and PTSD. Survey answers were not always clearly defined, particularly among individuals who used ketamine in multiple forms for multiple indications, but it largely appears that non-parenteral forms of ketamine were not associated with serious adverse effects.

In retrospect, survey design carries limitations in interpretation of responses. The survey did not distinguish its safety questions separately for each type of ketamine formulation, and many prescribers used more than one form of ketamine. For example, while only 3/45 respondents reported that no monitoring was necessary during administration, another 10 also reported home use in a non-medical setting. Use of other forms of ketamine by these 10 prescribers precludes clear understanding of their opinions on monitoring for home use.

One significant controversy regarding home use of ketamine is in regards to potential for ketamine abuse and diversion. Two previous reviews [30,31] and one retrospective survey [20] attempted to evaluate risk for abuse of ketamine when used as an antidepressant. The reviews noted no concerns in clinical studies beyond a few single case reports. The retrospective survey found that there were some patients who did”like” the effects and even had cravings for ketamine, but that overall risk appeared low. Our survey results appear consistent – few respondents noted cravings, drug-seeking behavior, or inappropriate use of ketamine, but notably none reported more than one of these things occurring at once. We noted that 10 respondents reported tolerance to ketamine, and this has been previously described in terms of tachyphylaxis to dissociative effects. While risk of ketamine abuse appears low, clinicians should be alert to signs of misuse, particularly with any at-home administration.

In respect to safety, only one respondent strictly used ketamine in a hospital setting, and this included IV treatments. All other respondents reported outpatient clinic use (with some prescribing take-home doses), supporting the idea that safety profile is favorable so as to not always require a hospital environment. Further, only 7/45 respondents felt that monitoring blood pressure was necessary. This finding is consistent with previous suggestions [32] that ketamine-induced hypertension is not of clinical importance unless symptoms of hypertensive emergency are present. Though hypertension over >180/110 was reported by almost 25% of respondents, duration was transient and most resolved spontaneously. Although antihypertensives were administered in some cases, there were no reports of symptomatic hypertension in the form of a hypertensive emergency, which according to previous reports on best practices of transient asymptomatic hypertension, would not medically necessitate treatment [22]. As such, from a safety standpoint, we would posit that blood pressure need not be routinely monitored, particularly during non-IV treatments, and that patients should be educated on symptoms of hypertensive emergency, which would necessitate emergent medical assessment such as calling an ambulance or proceeding to the emergency department. As there are no reports of hypertensive emergency with ketamine treatment in the literature, or in this survey of real world use, this would be an exceedingly rare effect, not dissimilar to hypertensive crisis with monoamine oxidase inhibitors, which patients routinely use at home. Perhaps more concerning were three responses for transient dysrhythmias. This would support the concept of completing a baseline ECG, particularly prior to parenteral administration of ketamine.

All medications have a range for effectiveness and a range for safety. While clinical trial data remains sparse for safe and efficacious doses for non-IV forms of ketamine for mental health indications, the data from this survey captures the real-life ranges that experienced providers use. In the absence of rigorous randomized controlled trials, the doses reported, combined with the reports of non-emergent adverse events, suggest that from a practical standpoint doses used are safe. That said, upper ranges for IV administration reported in this survey (2.5 mg/kg) must be considered with caution, as it is greater than the currently established safe and efficacious doses for depression. Of note, several providers indicated using ketamine for anesthesia or pain control, and these are likely the cases where advanced safety measures and increased monitoring is required as the higher range doses approach the anesthetic range for ketamine. Given the current body of evidence [31], it would be prudent to restrict IV ketamine doses to 0.5-1.0 mg/kg for treatment of depression.

The bioavailability of ketamine varies with different routes [33], which likely explains the wide variations in dosing between formulations. Approximate bioavailability between routes are as follows: IV 95-100%, IM/SC 90-95%, IN 30-50% SL 20-30%, PO 10-20%. With this in mind, doses of all parenteral forms could be comparable, and it could be considered that all parenteral routes of ketamine could carry similar efficacy to the evidence-based 0.5-1.0 mg/kg dosing of IV ketamine. This was seen in weight-based dosing of IM ketamine, which was in the range of 0.5-1.5 mg/kg, and it seems that the absolute dosing of 20-150 mg may align somewhat with this. Translating bioavailability to similar dosing for IN, SL and PO formulations is more challenging, but a recent community case series reported use of dose adjustments based on relative bioavailability; IN dosing 2-3 times that of IV and SL dosing 3-5 times that of IV [29].

A number of respondents commented on home use of ketamine without reports of significant adverse events. This is in contrast to recent warnings from the United States Food and Drug Administration (FDA) cautioning against use of home ketamine. When this is considered in the context of the clinical experience of these respondents, it suggests warnings do not align with the level of medical risk. This would suggest potential stigmatization regarding the dangerousness of ketamine in psychiatry. Similarly, safety measures in place were reported more often than felt “necessary,” suggesting that respondents have these measures in place only to satisfy regulatory bodies rather than reflective of medical risk. This variability speaks to the concept that due to ketamine’s anesthetic action, its safety profile is variable based on dosing and route. It is most likely that the sub-anesthetic, non-parenteral doses do not require the same safety measures in place as necessary in ketamine anesthesia. Many of the traditional safety practices and infrastructure that some clinics still employ may be based on the clinical context of anesthesia, which uses higher ketamine doses.

Interestingly, there was one response for aggressive behavior, lasting 25 minutes and requiring benzodiazepines with restraints. As this study included anesthetic indications, and these reactions have not been reported elsewhere in the psychiatric ketamine literature, we would postulate that this was in the setting of ketamine anesthesia as patients can experience emergence agitation as the anesthetic abates [34]. There was one response for prolonged trauma flashbacks, lasting 30 minutes and resolving spontaneously with psychological support. This would indicate a need to exercise caution in using ketamine in individuals with trauma histories and psychological support may be required. Overall, the majority of adverse effects were brief, not serious, and few required intervention, further strengthening the real world evidence for ketamine’s safety profile. Serious adverse events were not reported by prescribers of only non-parenteral ketamine.

A significant limitation to interpreting results is the low response rate to the survey. The multidisciplinary professional interest group providing the foundation of our convenience sample included approximately 250 members. While it is not clear how many of this group were prescribers, notably only 45 respondents completed the survey, which limits generalizability of results. However, of the respondents, over 80% had 10 or more years of experience prescribing ketamine, with a large subset having more than 20 years of experience, and an overall average of 5 years. Though the sample was small for the group targeted, it appears that the group of respondents had a valuable level of experience to share. It is possible that newer prescribers were many of the non-respondents. Nevertheless, the experienced population answering this survey brings validity to the responses, as the survey elicits years of ketamine prescribing experience, rather than solely from a collection of new prescribers.

To be included in the survey, respondents had to endorse using ketamine for a mental health indication. However, some respondents also used ketamine for anesthesia and/or pain indications and questions surrounding dosing, safety measures and adverse effects did not clearly distinguish different indications. As a result, it is important that reported doses are not interpreted as effective doses for any given condition, but the fact they are being used speaks to a level of safety and comfort among prescribers.

Also, the survey did not distinguish among use for pure pharmacotherapy versus ketamine-assisted psychotherapy practices for mental health indications, which would typically require psychoactive effects and dissociation, and therefore are more likely to be associated with higher dose ranges of any formulation. Some KAP practices by default would also lead to continuous observation as the therapist may be present during the ketamine administration. In these cases, the therapist’s presence may not be out of concern for safety, but simply part of the therapeutic process. However, this remains speculation as another significant source of limitation was due to question design and response collection. To promote breadth of responses, most questions allowed short answers and free text responses. While using free text did provide flexibility and nuance in some cases, it also made some responses challenging to interpret, both individually and within broader analyses. Certain responses had to be omitted from categorical comparisons due to uninterpretable or missing data. However, even with these limitations, this survey provided useful data on real world use of ketamine. Reports on doses along with the fact that there were no advanced safety measures required in any case speaks to a level of safety with ketamine used for mental health indications, particularly with non parenteral formulations.

## Conclusion

This survey provided the perspective of 45 different providers with more than 1000 patient encounters over two countries. It would appear that ketamine use is currently extremely variable among providers, both in route and dosing, though ranges appear relatively consistent with approximated bioequivalent doses of the evidence-based IV doses for TRD [29]. This survey did not tease out differences in modality and dose per indication but looked at data as a gestalt. While interpretation is limited, information gained regarding doses that clinicians find beneficial and safe in their practices can help with selecting doses for future RCTs and other studies on ketamine for psychiatric indications. We would posit that this survey supports a safety profile for ketamine in that no serious adverse events requiring advanced measures occurred in any dosing or modality. The presence of common side effects and potentially concerning issues like bladder irritation, dysrhythmia, and misuse serves to remind that ketamine is not entirely benign and certainly may carry adverse effects in some cases, such as with long-term use or in the absence of a full medical workup.

Providers are advised to carefully consider the context of ketamine prescribing including doses, route, and individual patient factors when deciding the level of monitoring that is clinically required. This survey suggests that strict regulation of ketamine or universal mandates for monitoring are not necessary, but instead should be placed in the clinical decision-making of the provider as each situation may differ. Box 3 reviews our recommendations based on this survey and integration of current literature. Clinicians should continue to weigh potential risks, access, and potential benefits to patients in their decision making.

**Box 3. Recommendations**

-Non parenteral ketamine carries a favorable risk and side effect profile at doses reported.
- Abuse risk of ketamine appears low, but be alert for signs of misuse, particularly with take-home doses
- Monitoring in a clinical setting may not be required, particularly for non parenteral forms of ketamine.
- When monitored in a clinical setting, patients and staff should be educated on symptoms of hypertensive emergencies, and respond where appropriate rather than routine reatment of elevated blood pressures
- A baseline ECG should be considered, particularly for parenteral administration.
- In regards to dosing, restrict IV ketamine doses to 0.5-1.0mg/kg, consider using intranasal dosing 2-3 times patient’s weight-based IV dosing, and using SL dosing 3-5 times patient’s weight-based IV dosing.
- Use caution with ketamine treatment in those with trauma histories,and be aware that psychological support may be required.

## Supporting information

S1 Appendix Survey questions. (DOCX)

S1 Data Unedited data from respondents of the survey. (XLSX)
