# Supplementary material for: Ketamine for mental health - A naturalistic inventory of prescribing practices, safety, and adverse effects
Source: PLOS Ment Health. 2025 Apr 2;2(4):e0000215. doi: 10.1371/journal.pmen.0000215 (PMC12798408; doi:10.1371/journal.pmen.0000215)
Supplement: S1 Appendix — (DOCX) [file pmen.0000215.s001.docx]

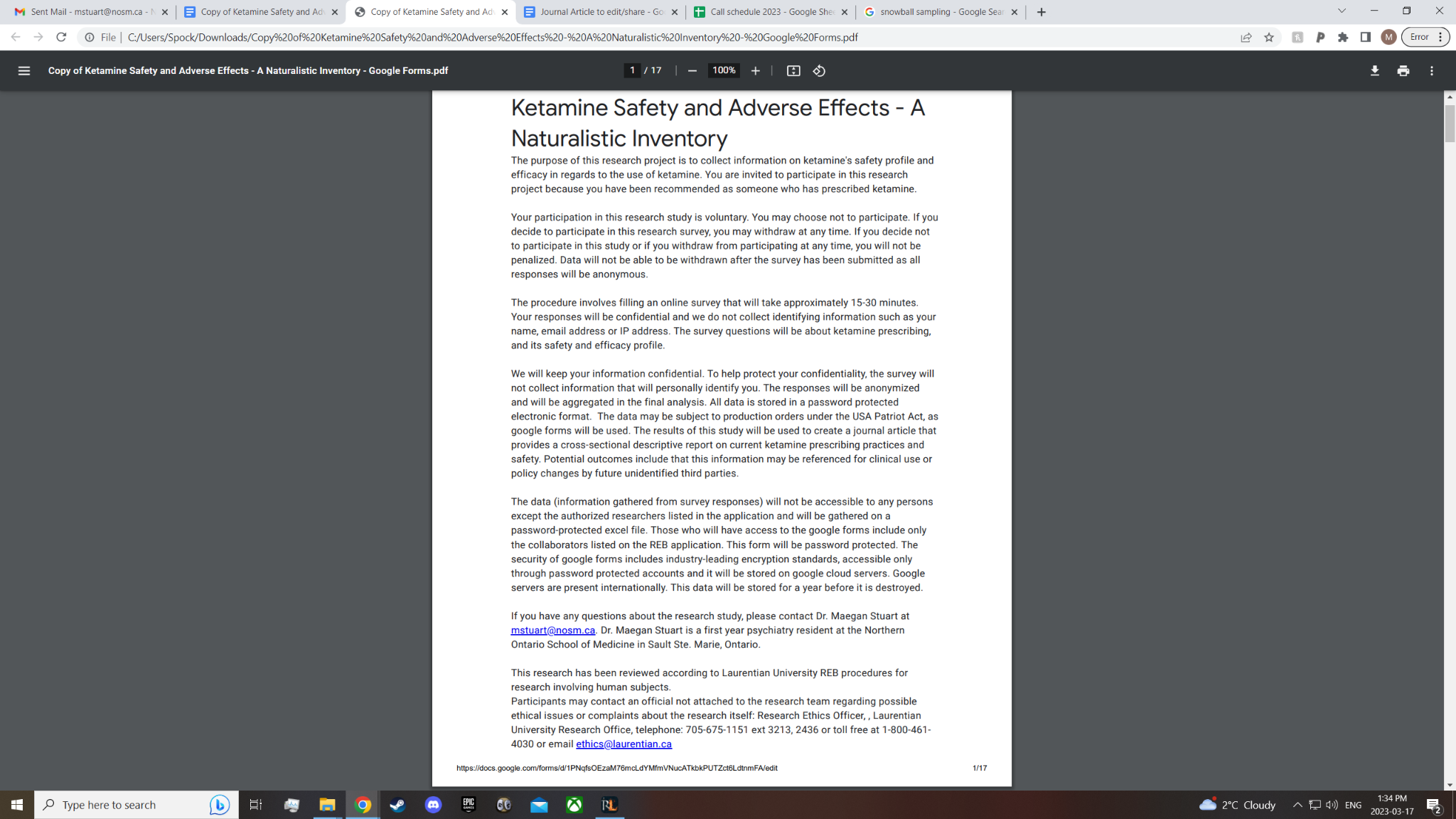

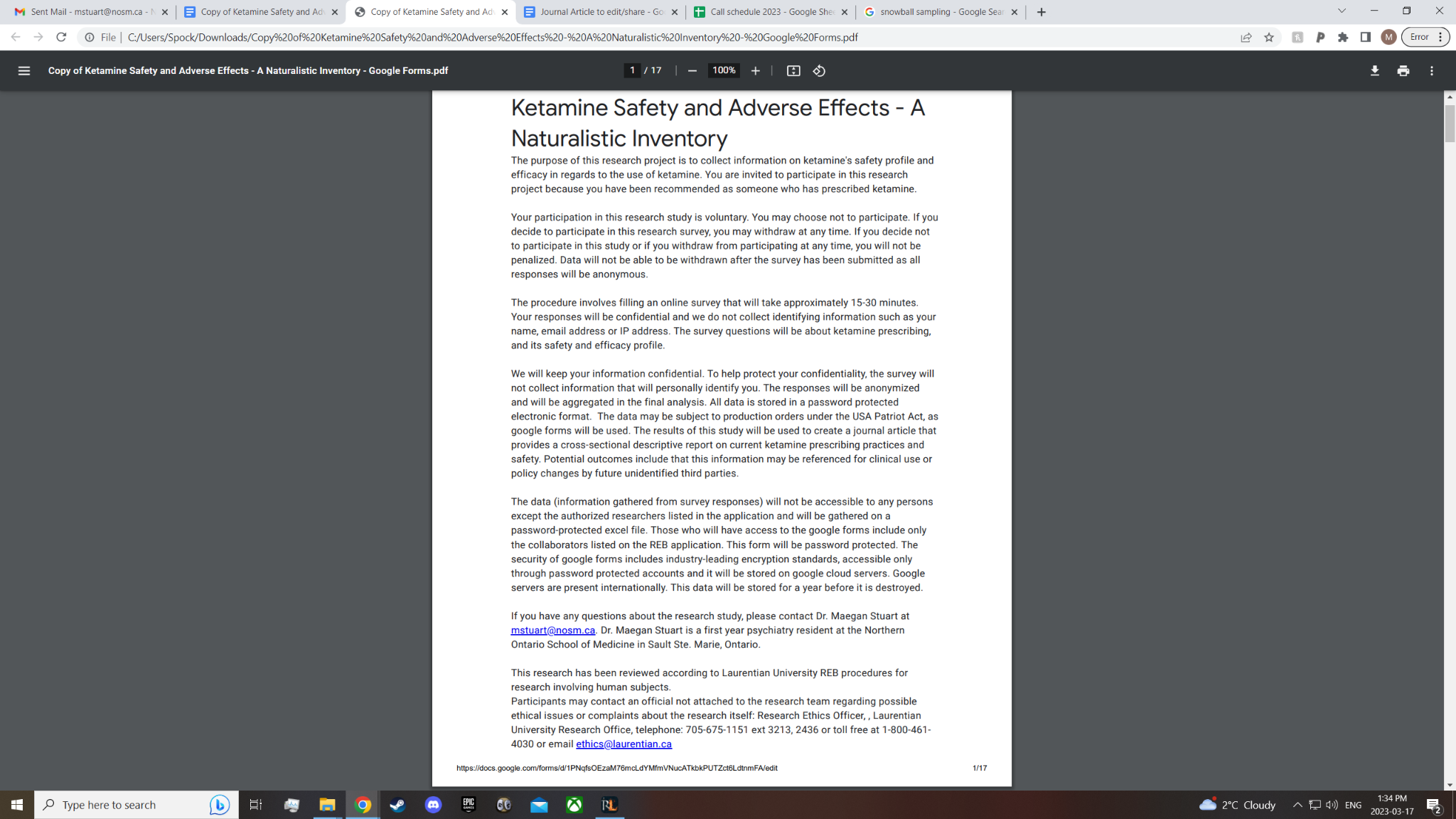


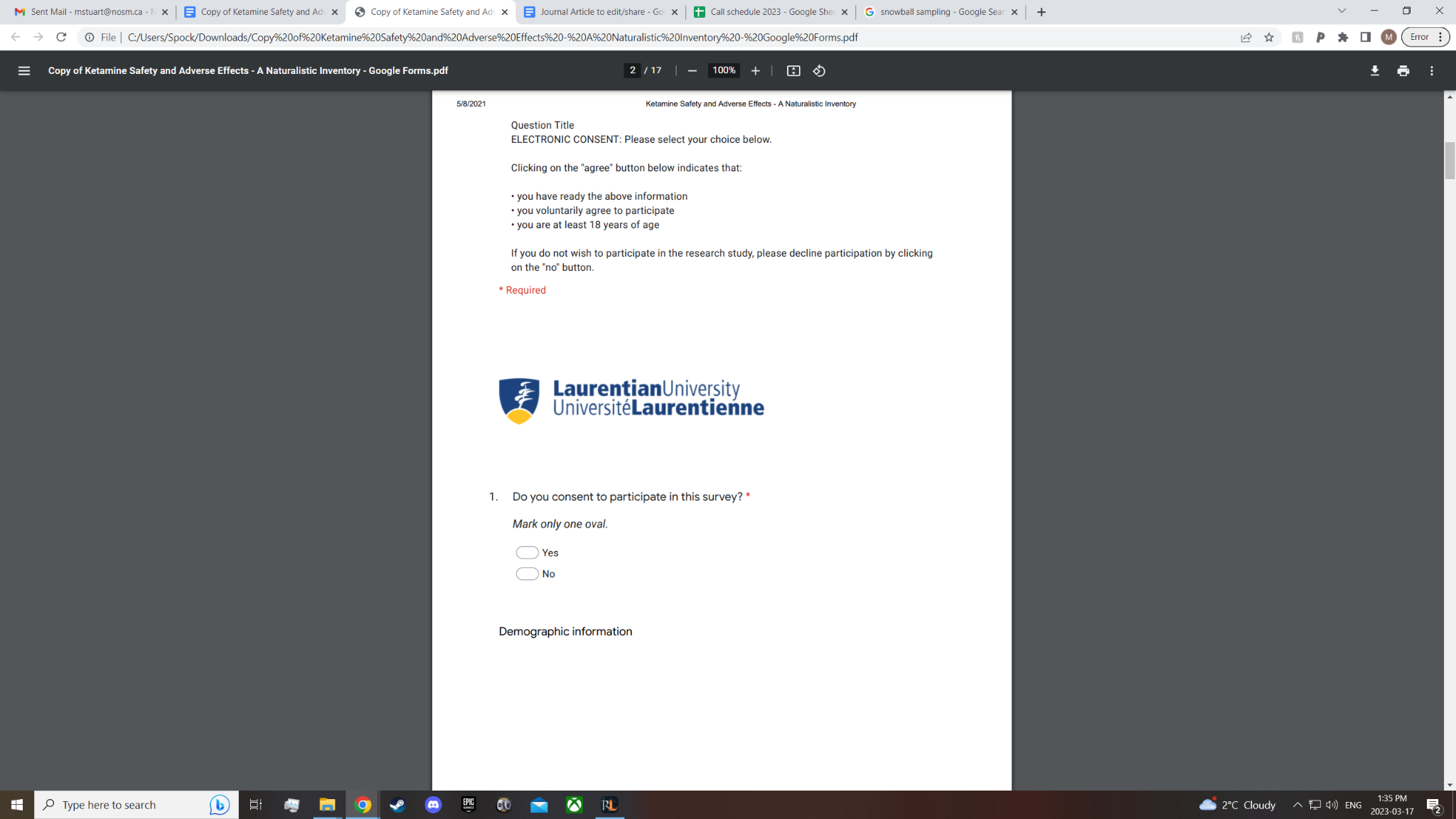

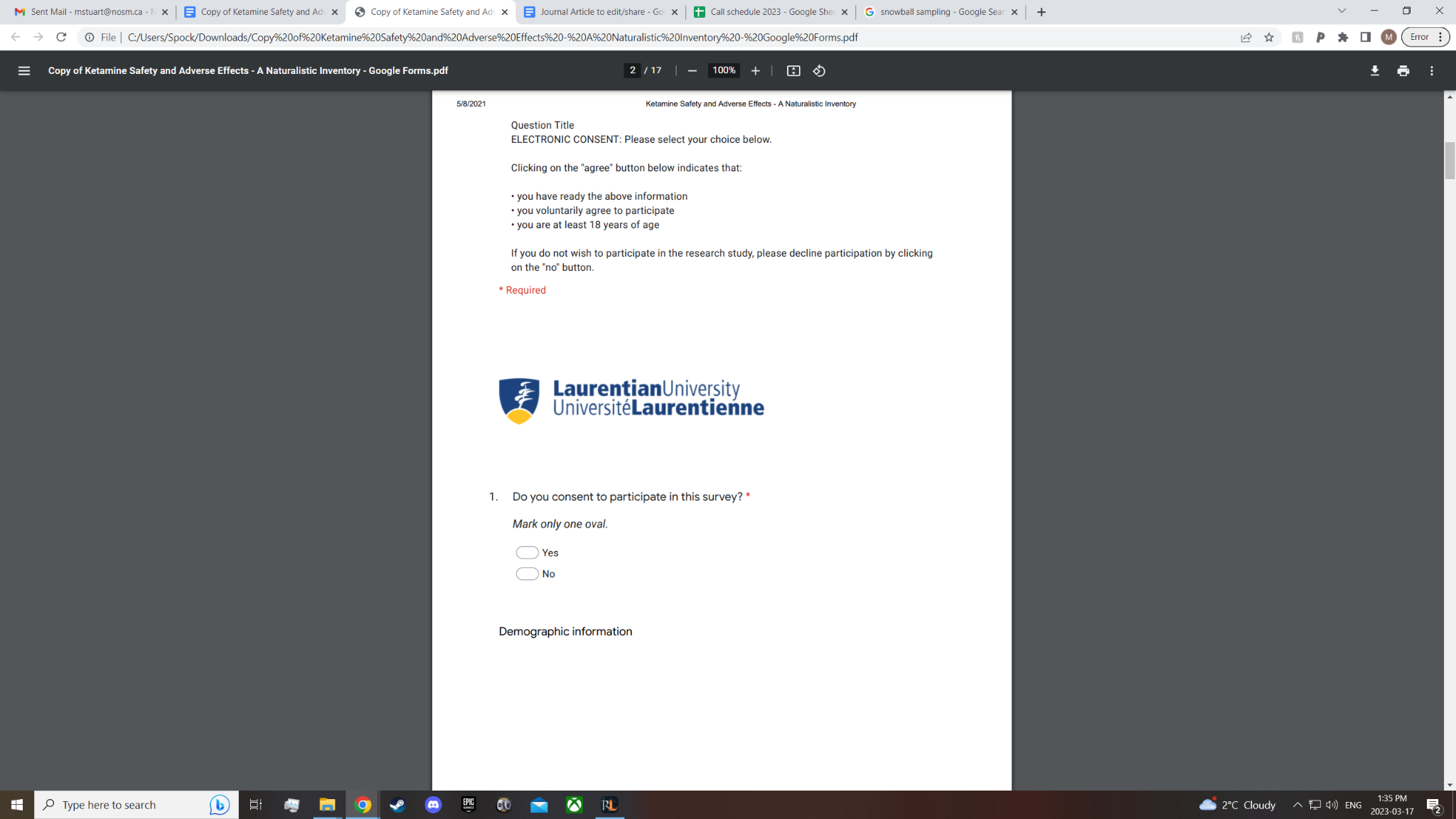


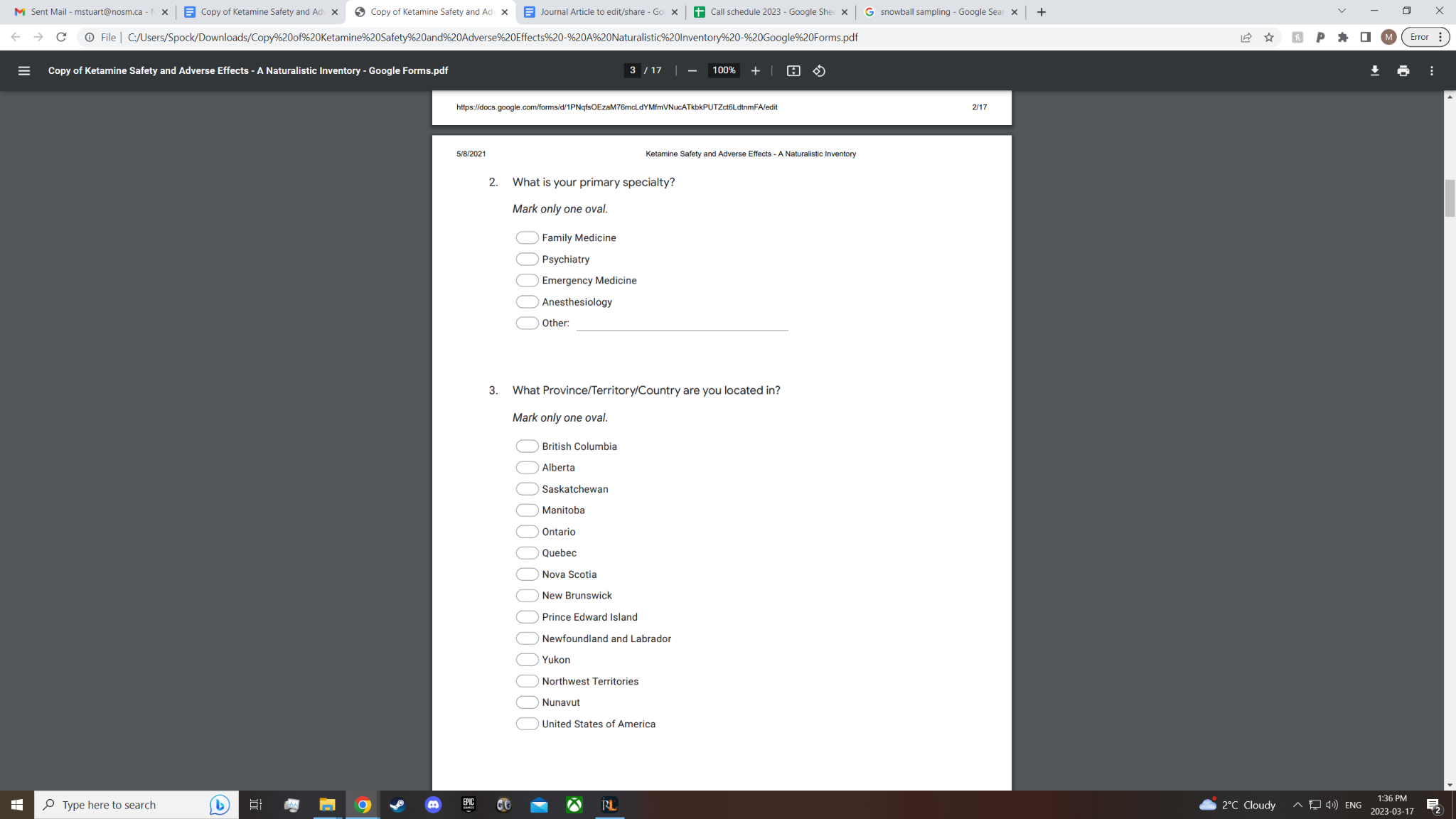


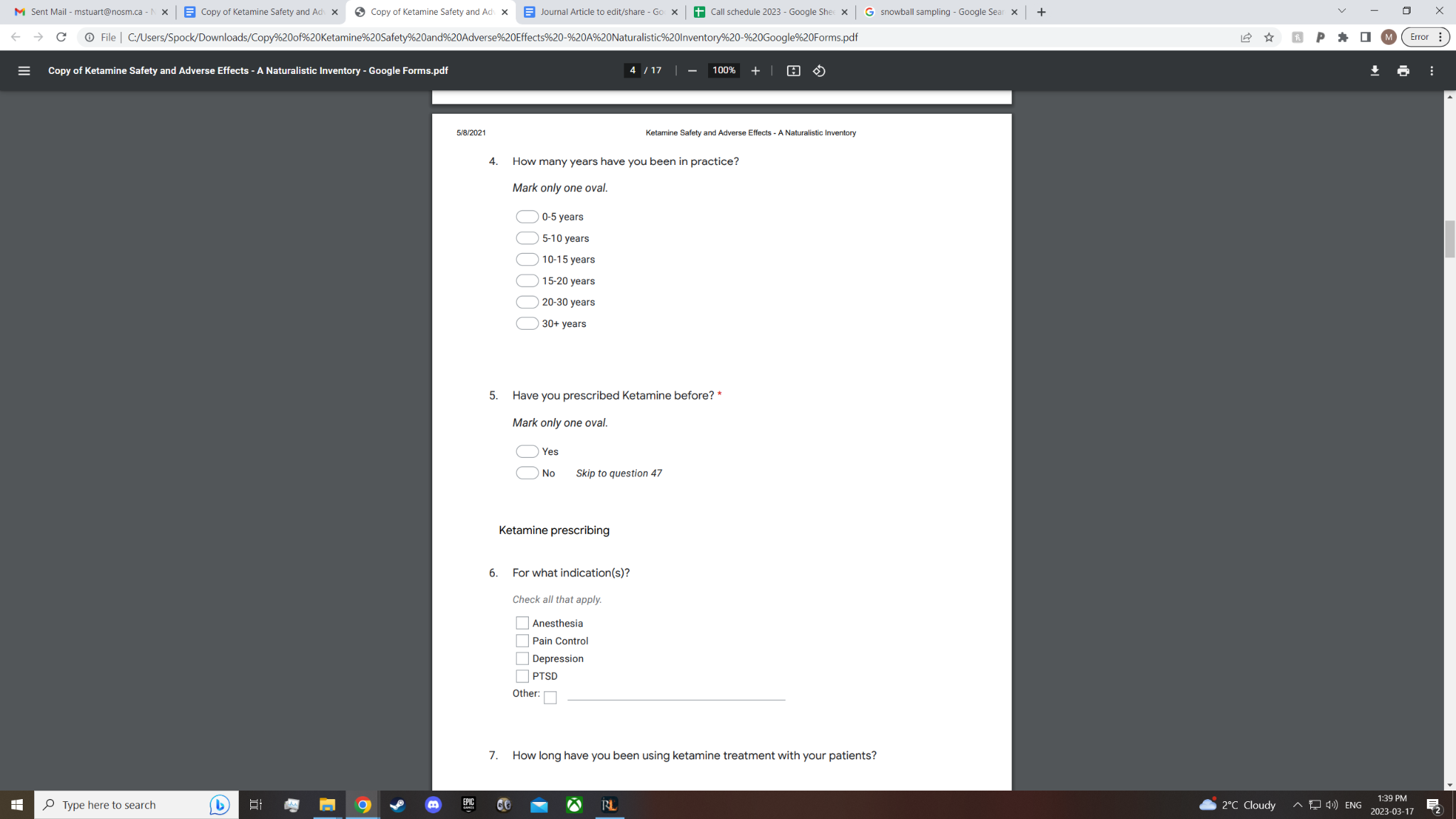


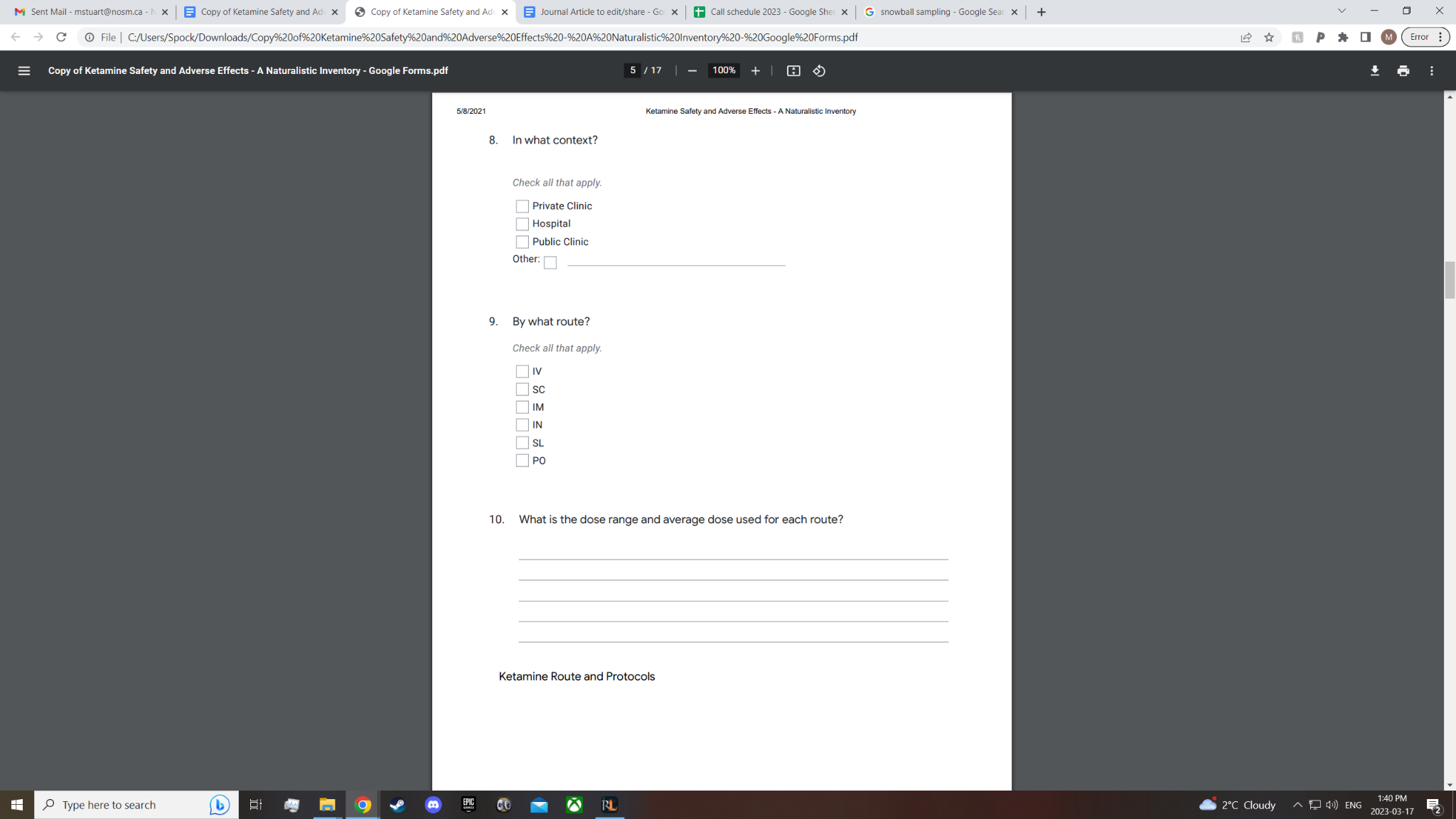


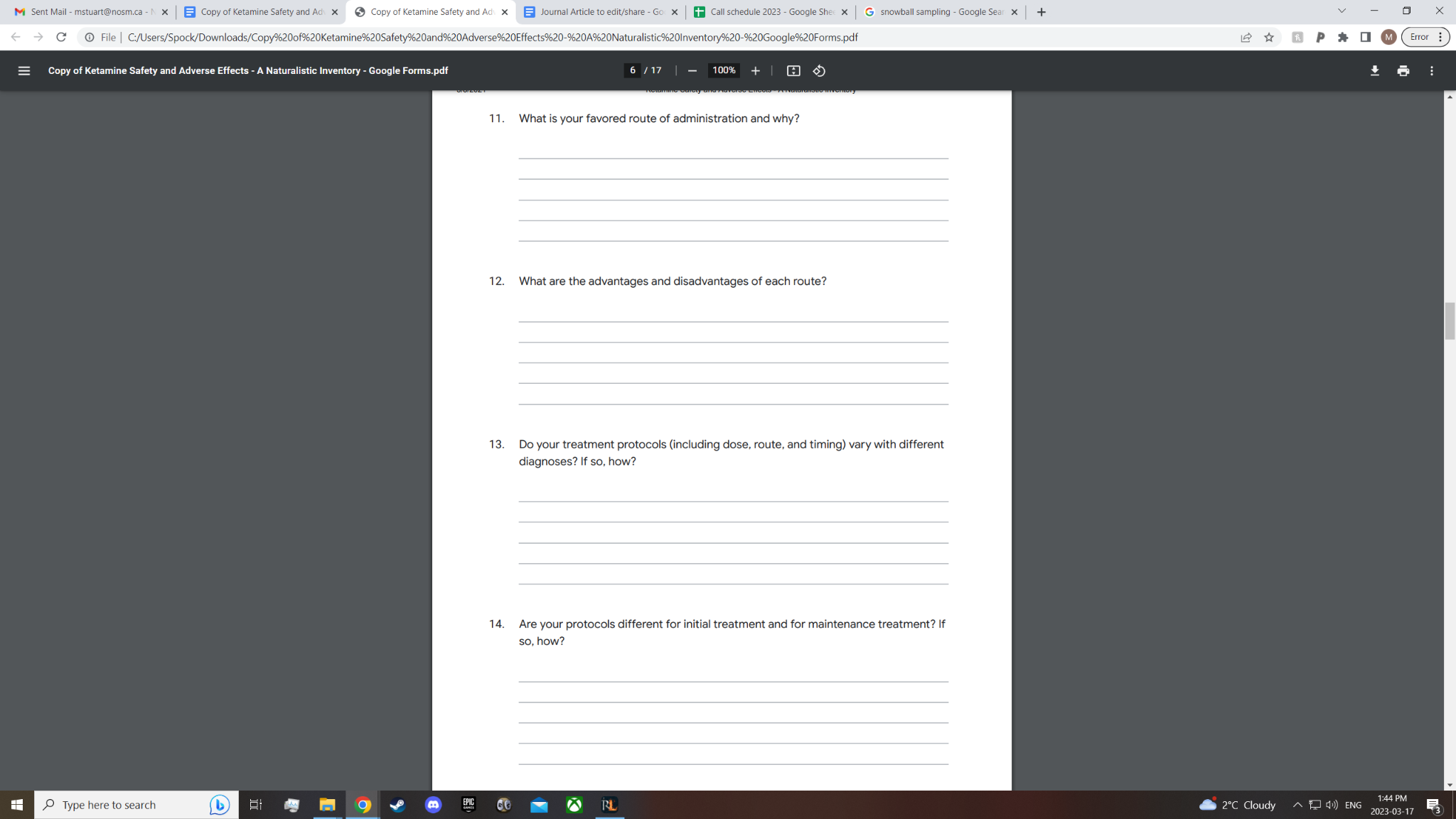


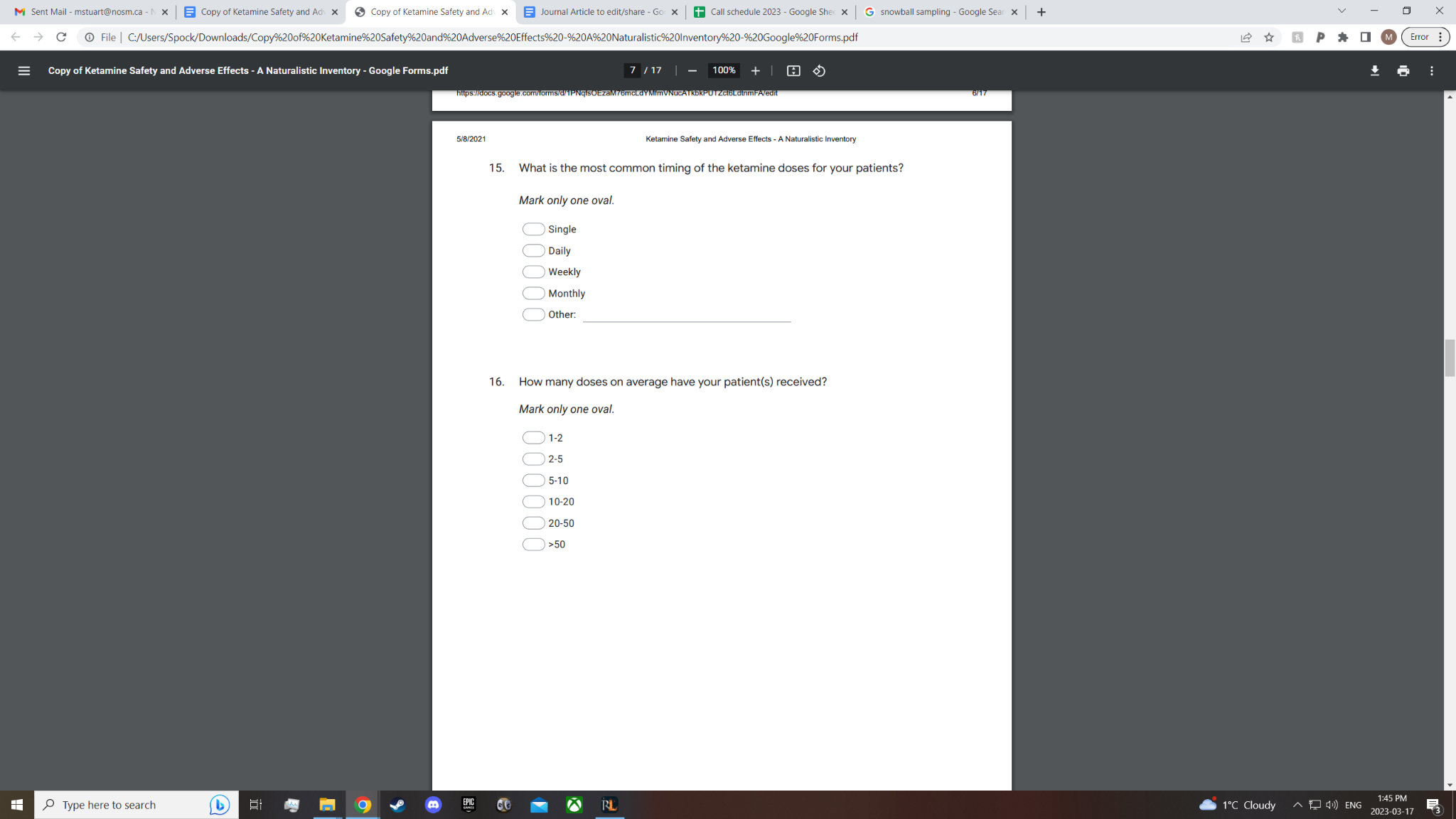


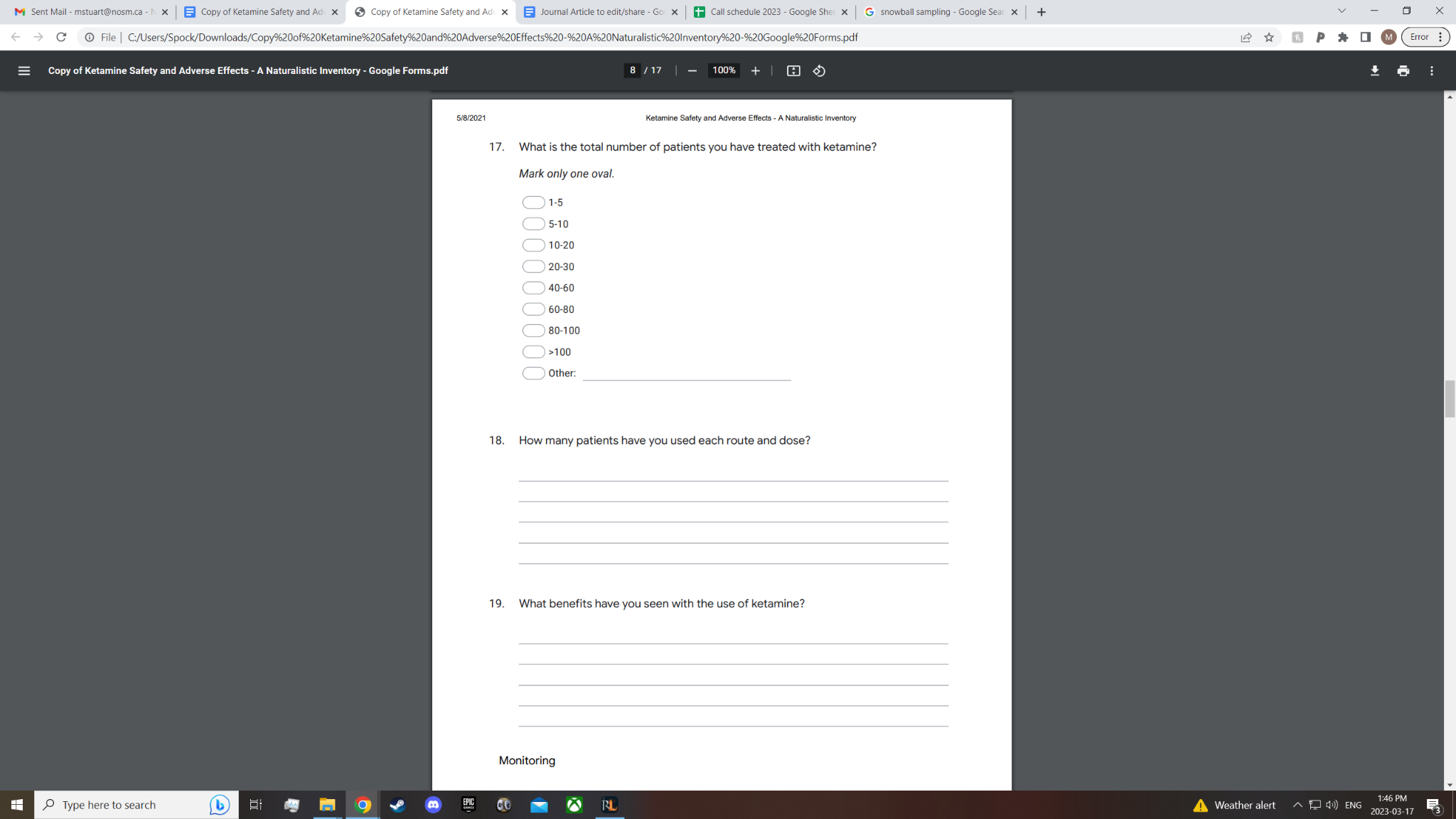


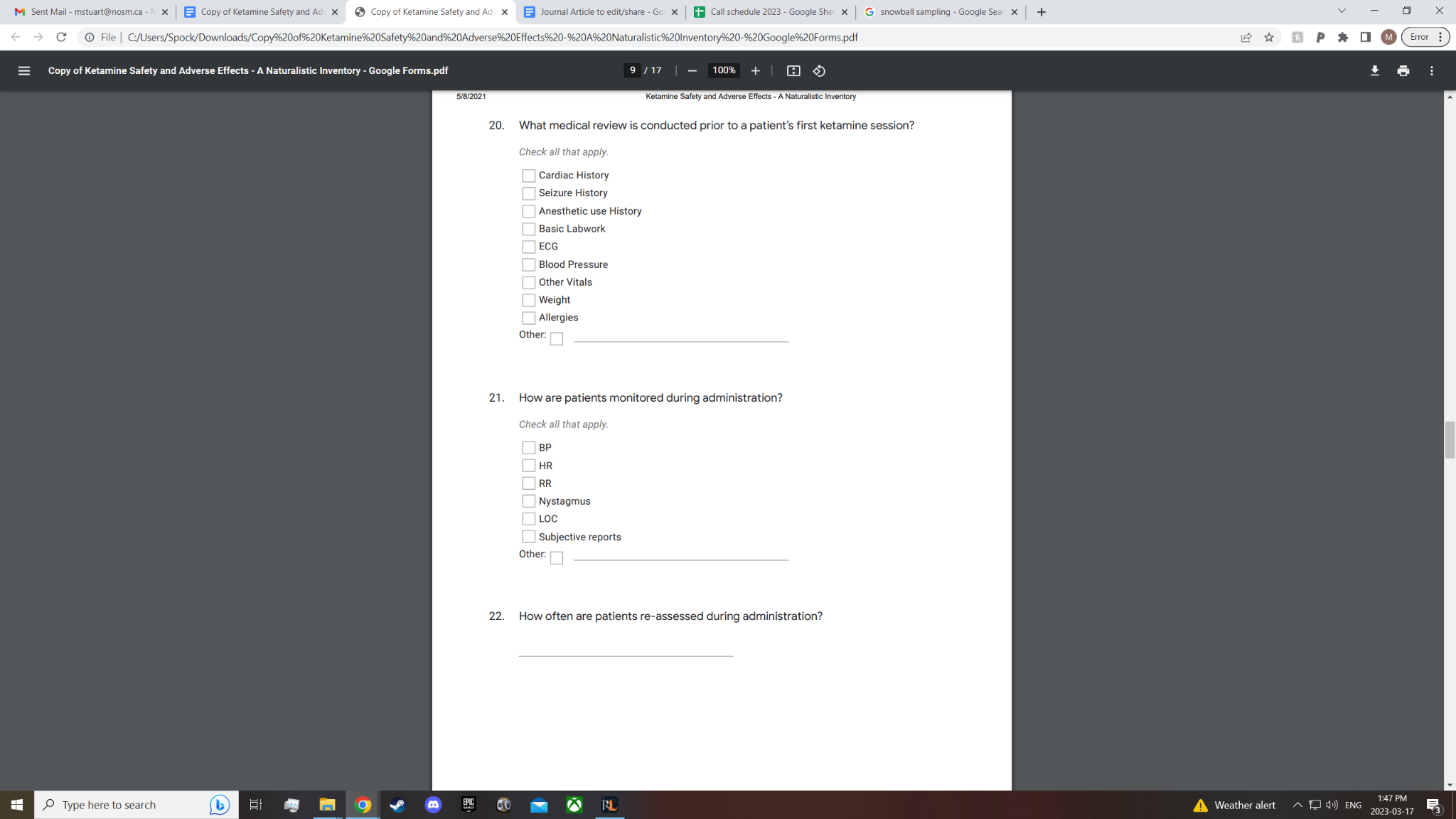


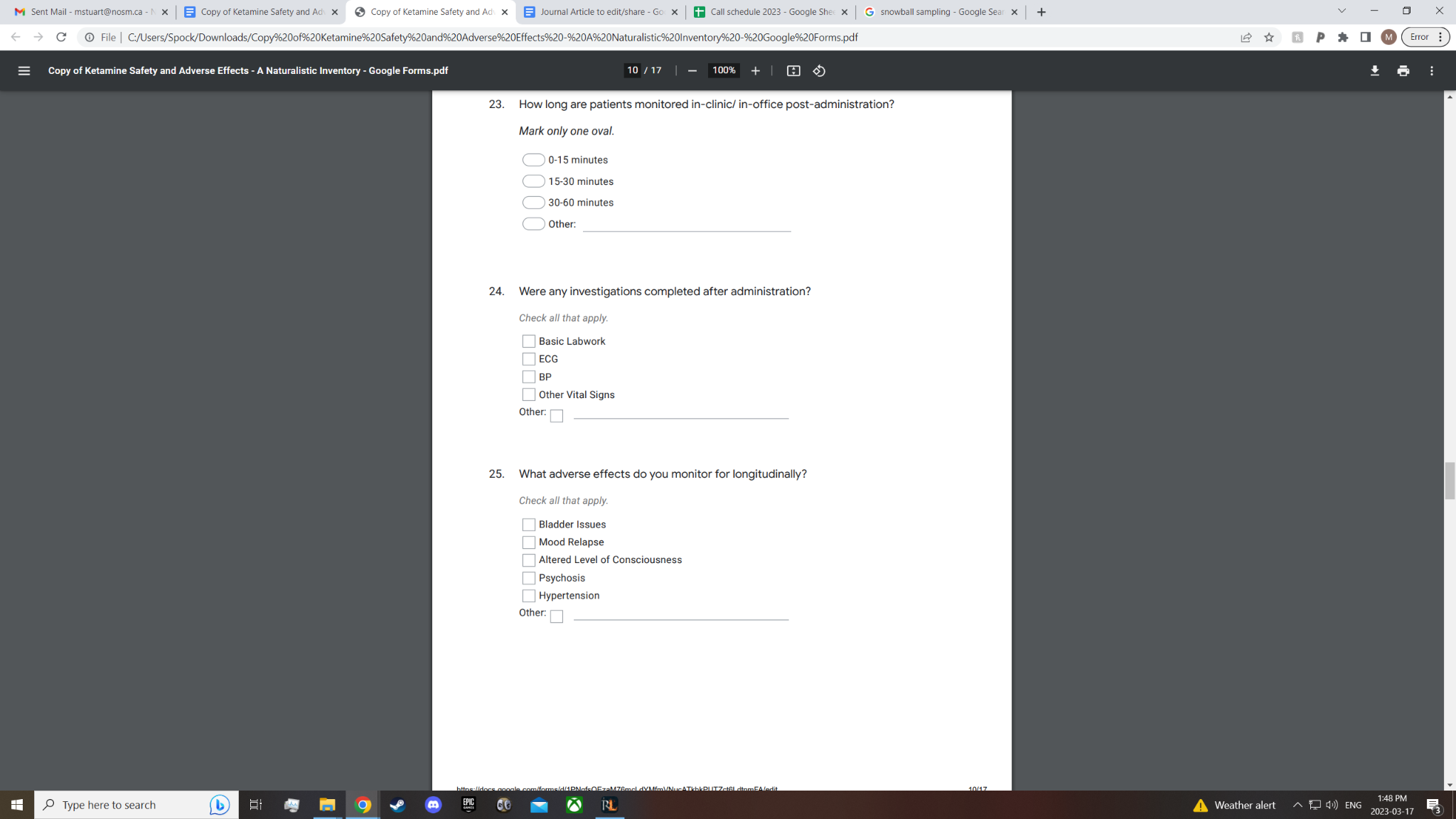


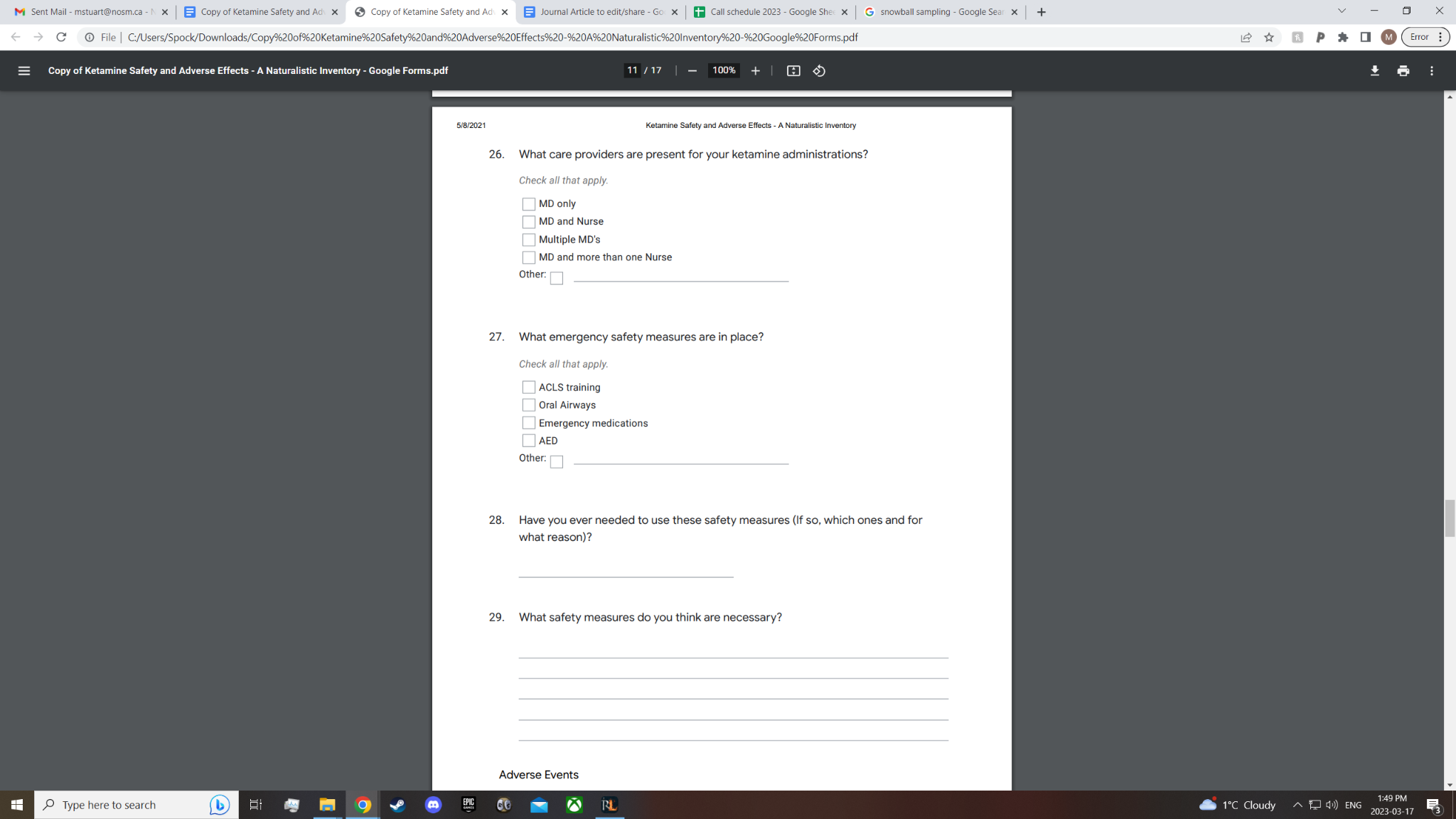


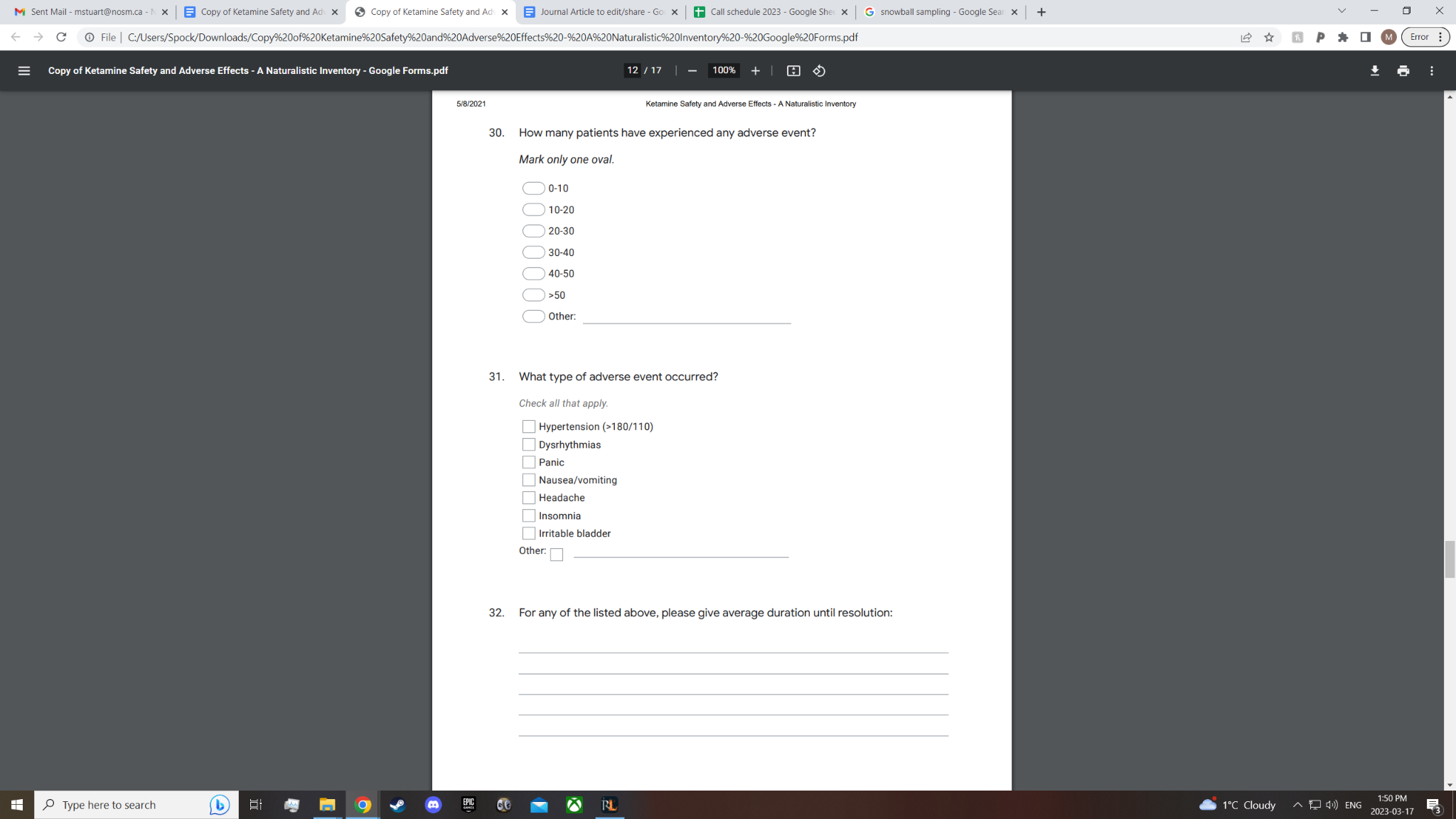


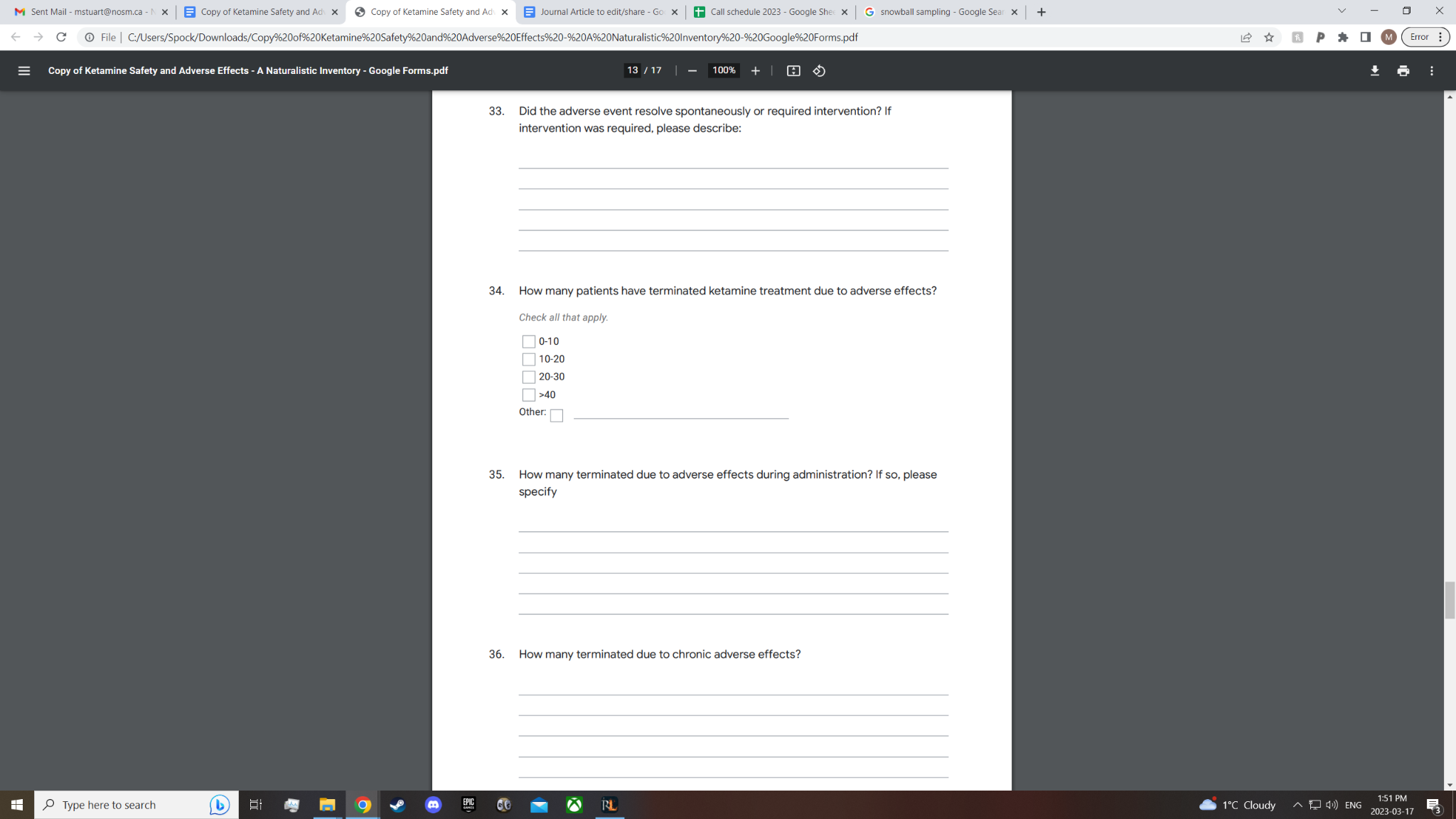


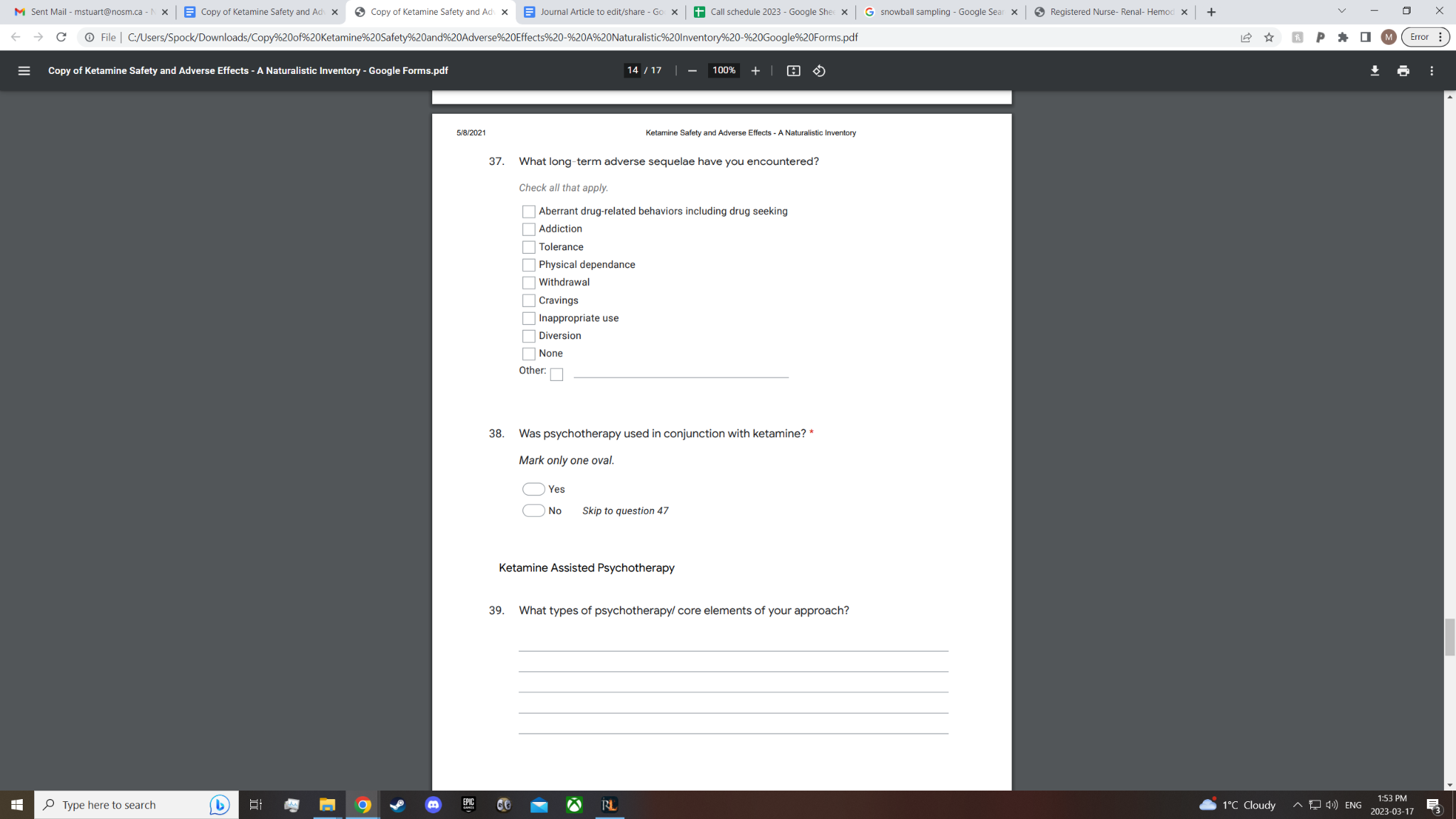


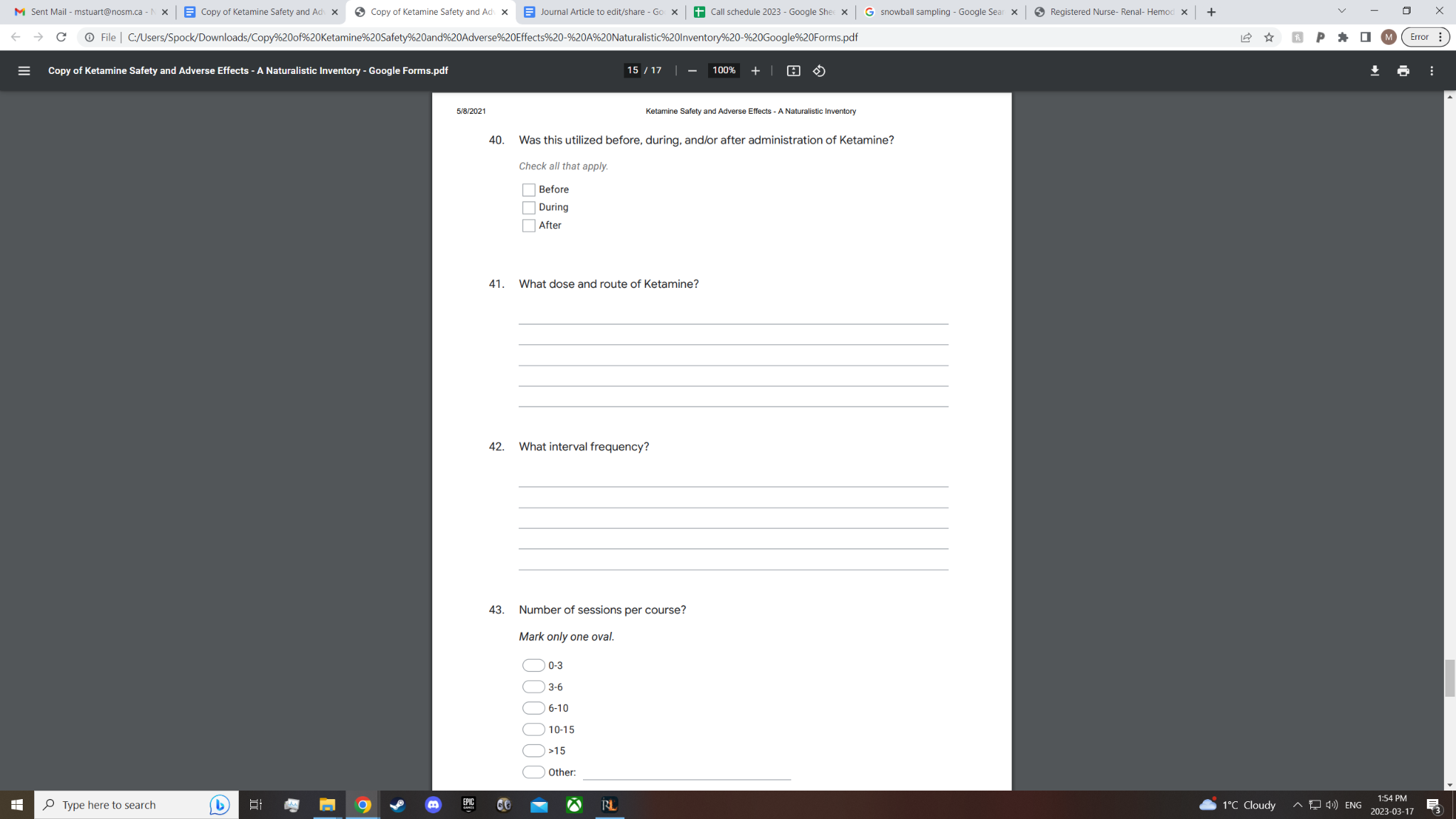


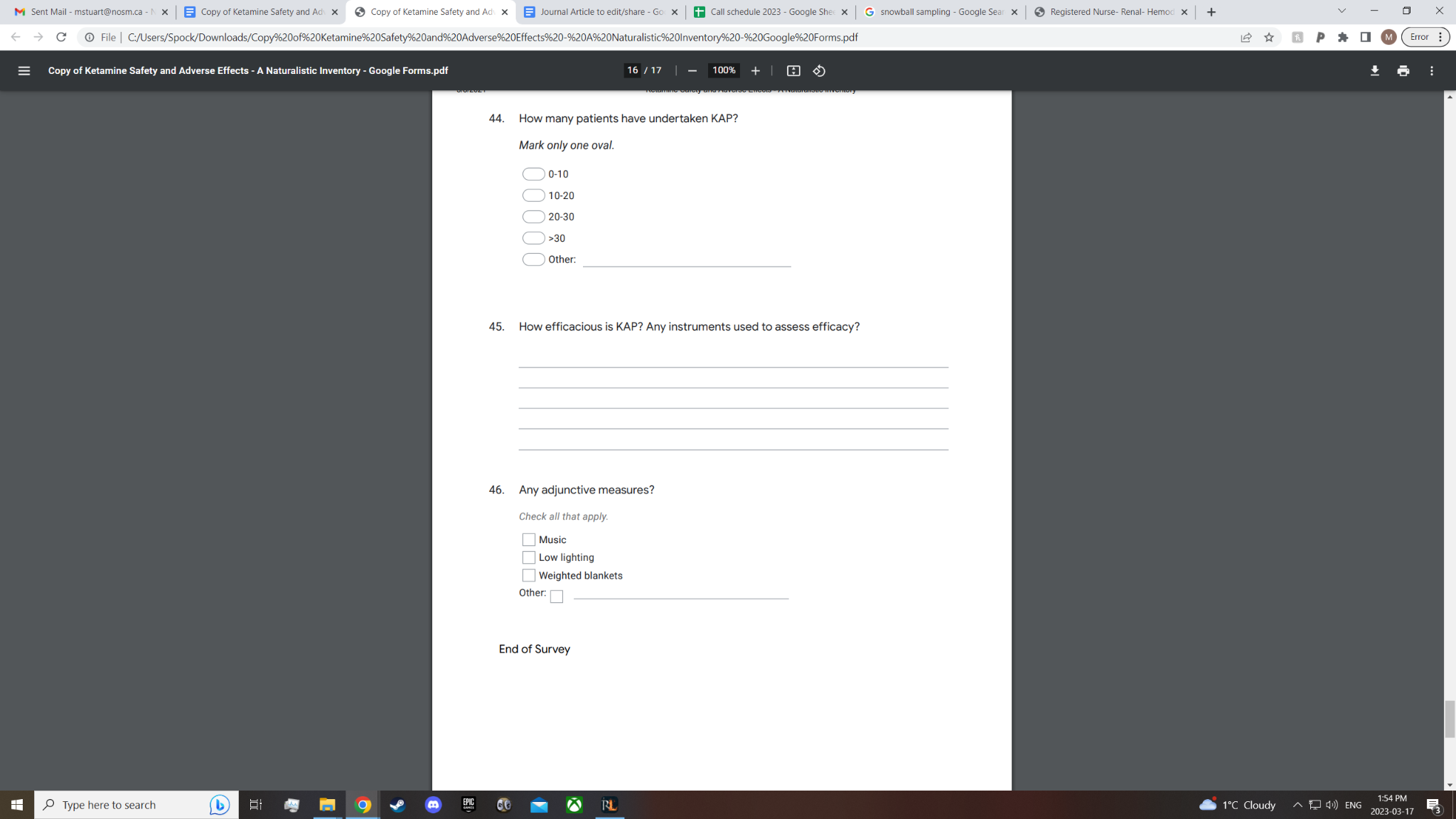


S1 Appendix: Survey questions that were provided to ketamine prescribers in Canada and the United States of America:
